# ACCEPTABILITY AND PRELIMINARY EFFICACY OF BE EPIC-VR TRAINING ON FRONTLINE HEALTH CARE WORKERS

**DOI:** 10.1093/geroni/igae098.0961

**Published:** 2024-12-31

**Authors:** Marie Savundranayagam, Grace Norris, Annette Schumann, Anya Sarma, Jennifer Campos, Joseph Orange

**Affiliations:** Western University, London, Ontario, Canada; Western University, London, Ontario, Canada; Western University, London, Ontario, Canada; Western University, London, Ontario, Canada; The KITE Research Institute, University Health Network, Toronto, Ontario, Canada; Western University, London, Ontario, Canada

## Abstract

Be EPIC-VR is a person-centered communication (PCC) training program designed for healthcare providers working in dementia care. It is the first virtual reality (VR) program to use conversational artificial intelligence to train users to communicate with avatars depicting persons living with dementia (PLWD). The current study examined the acceptability and preliminary efficacy of Be EPIC-VR training. Participants included eight personal support workers from four home care and long-term care settings. Focus groups were conducted both immediately after VR sessions and after completing the Be EPIC-VR training program. Data analyses used framework analysis. The theme, relevant training supporting learning, reflected the acceptability of Be EPIC-VR. Be EPIC-VR’s innovative design facilitated significant learning gains, highlighting the benefits of experiential design, accessibility of training components, relevance regardless of career level, and group feedback on learning outcomes. The theme supporting preliminary efficacy was applying newly learned knowledge and skills with PLWD. Four subthemes emerged that mapped onto Be EPIC-VR’s foci. First, participants used the cues from the environment when interacting with PLWD. Second, participants, including those with English as a second language, reported applying PCC strategies which helped in understanding PLWD’s needs and addressing care refusal. Third, they noted an increase in self-efficacy in dementia care, which strengthened relationships with PLWD. Finally, they reported incorporating the preferences of PLWD during care interactions. Be EPIC-VR training emerges as a promising tool for enhancing skills of personal support workers, suggesting that immersive, VR-based training programs can foster empathetic, knowledgeable, and person-centered care approaches.

